# Using bioinformatics analysis to screen abnormal methylated differentially expressed hub genes of Kawasaki disease and construct diagnostic model

**DOI:** 10.1016/j.heliyon.2022.e11905

**Published:** 2022-11-25

**Authors:** Hongxiao Sun, Changying Liu, Xu Zhang, Panpan Liu, Zhanhui Du, Gang Luo, Silin Pan

**Affiliations:** a Heart Center, Women and Children’s Hospital, Qingdao University, 266034 Qingdao, China; b Rehabilitation Medicine Department, Women and Children’s Hospital, Qingdao University, 266034 Qingdao, China; c Anesthesiology Department, Women and Children’s Hospital, Qingdao University, 266034 Qingdao, China

**Keywords:** Differentially expressed genes, Gene expression omnibus, Kawasaki disease, Protein-protein interaction network, Random forest

## Abstract

**Objective:**

By using bioinformatics analysis, abnormal methylated differentially expressed genes (MDEGs) in Kawasaki disease (KD) were identified and a random forest diagnostic model for KD was established.

**Methods:**

The expression (GSE18606, GSE68004, GSE73461) and methylation (GSE109430) profiles was retrieved and download from Gene Expression Omnibus (GEO). We conducted enrichment analyses by using R software. In addition, we constructed a protein interaction network, and obtained 6 hub genes. We used expression profiles GSE100154 from GEO to verify the hub genes. Finally, we constructed a diagnostic model based on random forest.

**Results:**

We got a total of 55 MDEGs (43 hyper-methylated, low-expressing genes and 12 hypo-methylated, high-expressed genes). Six hub genes (CD2, IL2RB, IL7R, CD177, IL1RN, and MYL9) were identified by Cytoscape software. The area under curve (AUC) of the six hub genes was from 0.745 to 0.898, and the combined AUC was 0.967. The random forest diagnostic model showed that AUC was 0.901.

**Conclusion:**

The identification of 6 new hub genes improves our understanding of the molecular mechanism of KD, and the established model can be employed for accurate diagnosis and provide evidence for clinical diagnosis.

## 1 Introduction

Kawasaki disease (KD) is an acute self-limited disease, involving small and medium arteries, which with unknown etiology, also known as mucocutaneous lymph node syndrome [1]. KD is the most common acquired heart disease in developed countries, and the main age of onset is under 5 years old [2]. Nonspecific fever, bilateral bulbar conjunctival injection, changes of lips and oral cavity, rash, changes of peripheral extremities, and non-suppurative cervical lymphadenopathy are principal clinical features of KD [3]. The aim of treating KD in the acute phase is to reduce the damage caused by inflammation to the coronary artery wall and prevent coronary artery thrombosis. About 25% untreated KD patients developed coronary artery lesions (CALs), which can lead to acute myocardial infarction or even sudden death in severe cases [4]. At present, the diagnosis of KD mainly depends on clinical manifestations, without specific diagnostic indicators. Part of KD patients has atypical clinical manifestations, which are easy to miss diagnosis and delay treatment, and finally appear serious complications. Therefore, it is very important to find a new specific diagnostic method.

In recent years, genome-related technology developed rapidly, and bioinformatics analysis based on gene expression profiles has been widely used to explore the mechanism of disease and identify potential biomarkers, among which gene microarray is widely used, and related articles have been published extensively. Epigenetic regulatory mechanism is a key process in coordinating cellular processes and functions [5]. DNA methylation is an important part of epigenetic modification, which can regulate gene expression, and strengthen its connection with chromatin remodeling and histone modification [6]. Therefore, we integrated gene expression data with gene methylation data to obtain truly meaningful disease-related genes. In this study, the bioinformatics analysis was used to analysis data from the gene expression profiling microarrays (GSE18606, GSE68004, GSE73461) and gene methylation microarrays (GSE109430). Exploring the hub genes, signaling pathways, interaction networks associated with KD. We aimed to find new abnormal methylated differentially expressed genes (MDEGs) that can become biomarkers to specific diagnosis of KD, and construct a KD diagnostic model based on these hub genes.

## 2 Methods

### 2.1 Microarray data

Three gene expression profiling datasets (GSE18606 [7], GSE68004 [8], GSE73461 [9]), one gene methylation profiling dataset (GSE109430 [10]) were identified following a search of the Gene Expression Omnibus (GEO, https://www.ncbi.nlm.nih.gov/geo/) database. All the datasets were obtained from whole blood samples. GEO serves as a public database of genetic data, in which patient specimens have been ethically approved. Data in GEO is free for anyone to research and publish. As a result, our research is based on open and free data and is free of ethical issues and other conflicts of interest. Relevant ethical approvals and patient informed consent can be obtained from GEO (GSE18606: PMID: 20600450; GSE68004: PMID: 29813106; GSE73461: PMID: 30083721; GSE109430: PMID: 30382880).

GSE18606 contained 20 patients with KD and 9 healthy samples from GPL6480 (Agilent-014850 Whole Human Genome Microarray 4 × 44K G4112F). GSE68004 contained 89 patients with KD and 37 healthy samples from GPL10558 (Illumina HumanHT-12 V4.0 expression Beadchip). GSE73461 contained 78 patients with KD and 55 healthy samples from GPL10558. GSE109430 contained 12 patients with KD and 12 healthy samples from GPL13534 (Illumina HumanMethylation450 BeadChip). The data were cleaned using Limma package [11], Lumi package [12], and CHAMP package [13]. AnnoProbe package [14] was used to convert the probe number to gene symbol. All the analysis was processed in R software (4.1.0).

### 2.2 Data processing

Differentially expressed genes (DEGs) between KD and healthy samples were analyzed using the Limma package [11]. Genes were defined as DEGs if they met the cutoff criteria of adjusted P < 0.05 and |logFC| > 1.0. We got the list of full DEGs, the list of upregulated DEGs, and the list of downregulated DEGs in three expression profiling microarray datasets. The methylation profiling microarray dataset was analyzed to screen differentially methylated genes (DMGs) by using the CHAMP package [13]. Genes were defined as DMGs if they met the cutoff criteria of adjusted P < 0.05 and |δβ| > 0.2. We used the VennDiagram package to identify the overlapping DMGs and DEGs and generated the Venn diagrams. Meanwhile, we screened the expression data of the top 50 adjust P values of DEGs (|logFC| > 1.0) or DMGs (|δβ| > 0.2) in these four datasets, and applied Heatmap package to draw heatmaps.

### 2.3 Functional and pathway enrichment analyses

Gene Ontology (GO) enrichment analysis is the most widely used method to obtain gene function, including molecular function (MF), cellular component (CC), and biological process (BP). Kyoto Encyclopedia of Genes and Genomes (KEGG) is a database that retrieves information about genomic, chemical, and system functional. We collected the MDEGs and converted the gene symbol to enterz ID. The results of GO and KEGG enrichment analysis of MDEGs in this study were analyzed by using ClusterProfiler package [15]. We set the adjusted P-value cut-off to 1 × 10^−4^ and the Q value cutoff to 1 × 10^−4^.

### 2.4 PPI network construction and hub gene identification

We used the Search Tool for the Retrieval of Interacting Genes (STRING, https://www.string-db.org/) database to perform the protein-protein interactions (PPI) networks. In order to obtain possible protein correlation, MDEGs was retrieved in STRING database and PPI pairs with comprehensive score >0.15 were displayed. To select hub genes, we used all twelve ranking methods (MCC, DMNC, MNC, Degree, EPC, BottleN, EcCentricity, Closeness, Radiality, Betweenness, Stress, and Clustering Coefficient) in the Cytohubba plug-in of Cytoscape software and overlapped the top genes. Hypermethylation and down expression genes ranked top 10 genes, while, hypermethylation and down expression genes ranked top 5 genes. The first three MDEGs that appeared most times were selected as hub genes respectively. Finally, we used the MCODE plugin to obtain modules, and we used MCODE score >3, the number of nodes >3, to select defining modules.

### 2.5 Results validation

We used whole blood gene expression profiling datasets GSE100154 [16] from GPL6884 (Illumina HumanWG-6 v3.0 expression beadchip) as the validation dataset (The ethical approvals and patient informed consent can be obtained from PMID: 33377660, PMID: 32736569 and PMID: 34282143). The ggplot2 package was used to draw the box diagram of 6 hub genes. We also draw the receiver operating characteristic curve (ROC) by pROC package [17]. The area under curve (AUC) was used to evaluate the diagnostic value.

### 2.6 Establishment of diagnostic model

Random forest (RF) is a classifier with multiple decision trees [18], and has widely used of applications in bioinformatics. With 332 samples in all four gene expression datasets, we established a random forest model based on 6 hub genes by randomForset package [19]. All 332 samples were divided into training set and testing set according to 7:3, and obtained AUC, accuracy, sensitivity, specificity, and mean decrease Gini.

## 3 Results

### 3.1 Identification of abnormally MDEGs

**Figure 1:**
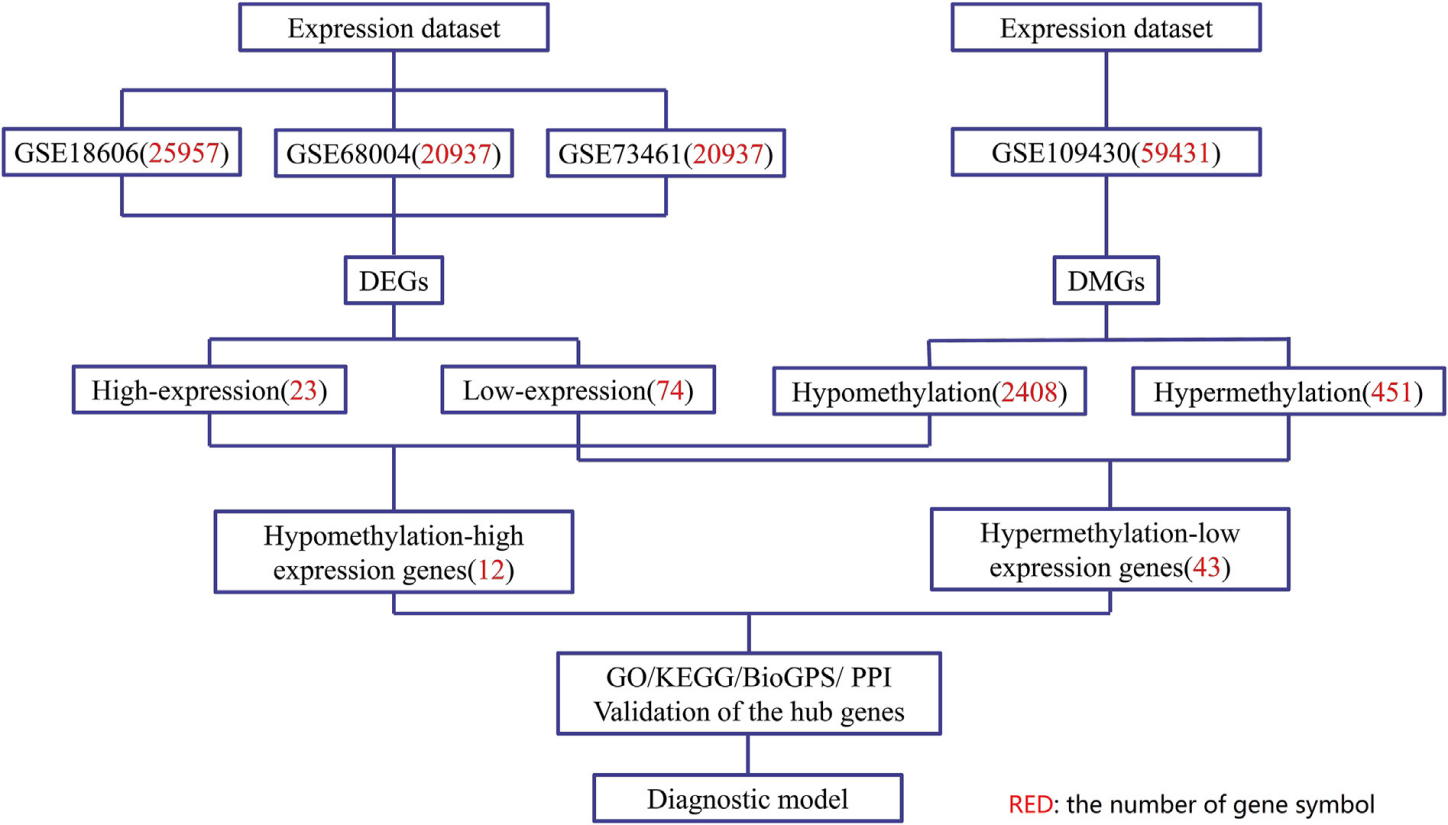
The flow chart of this study.

**Figure 2:**
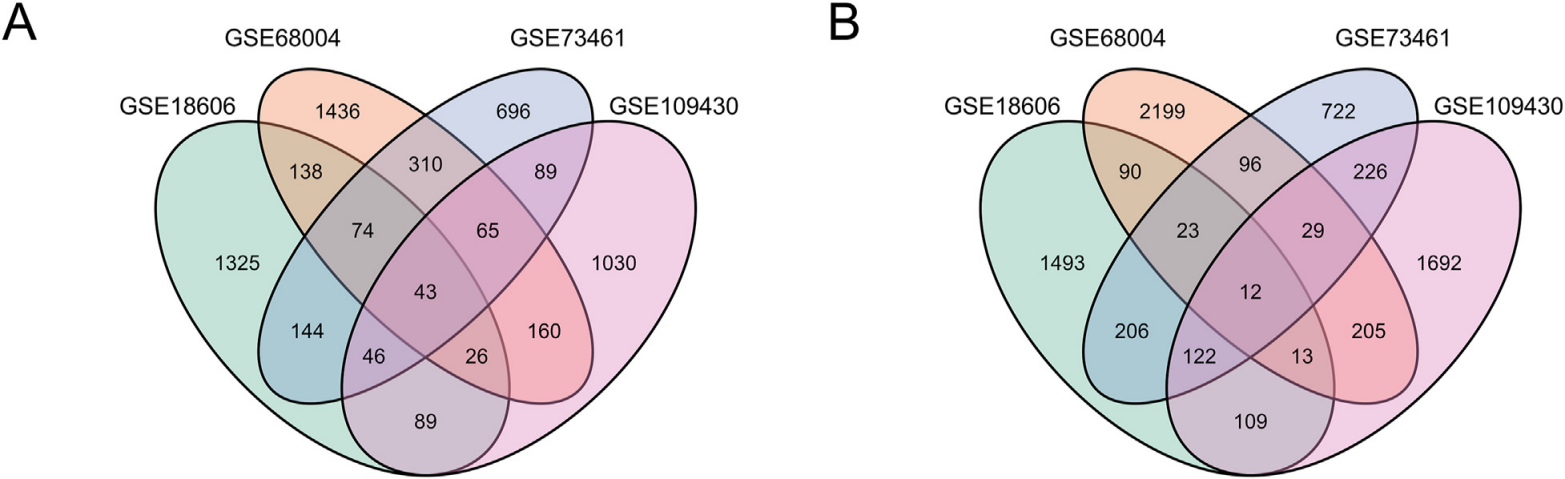
Identification of MDEGs in gene expression profiling datasets (GSE18606, GSE68004, GSE73461) and gene methylation profiling datasets (GSE109430). (A) Hypermethylation and low expression genes. (B) Hypomethylation and high expression genes.

**Figure 3:**
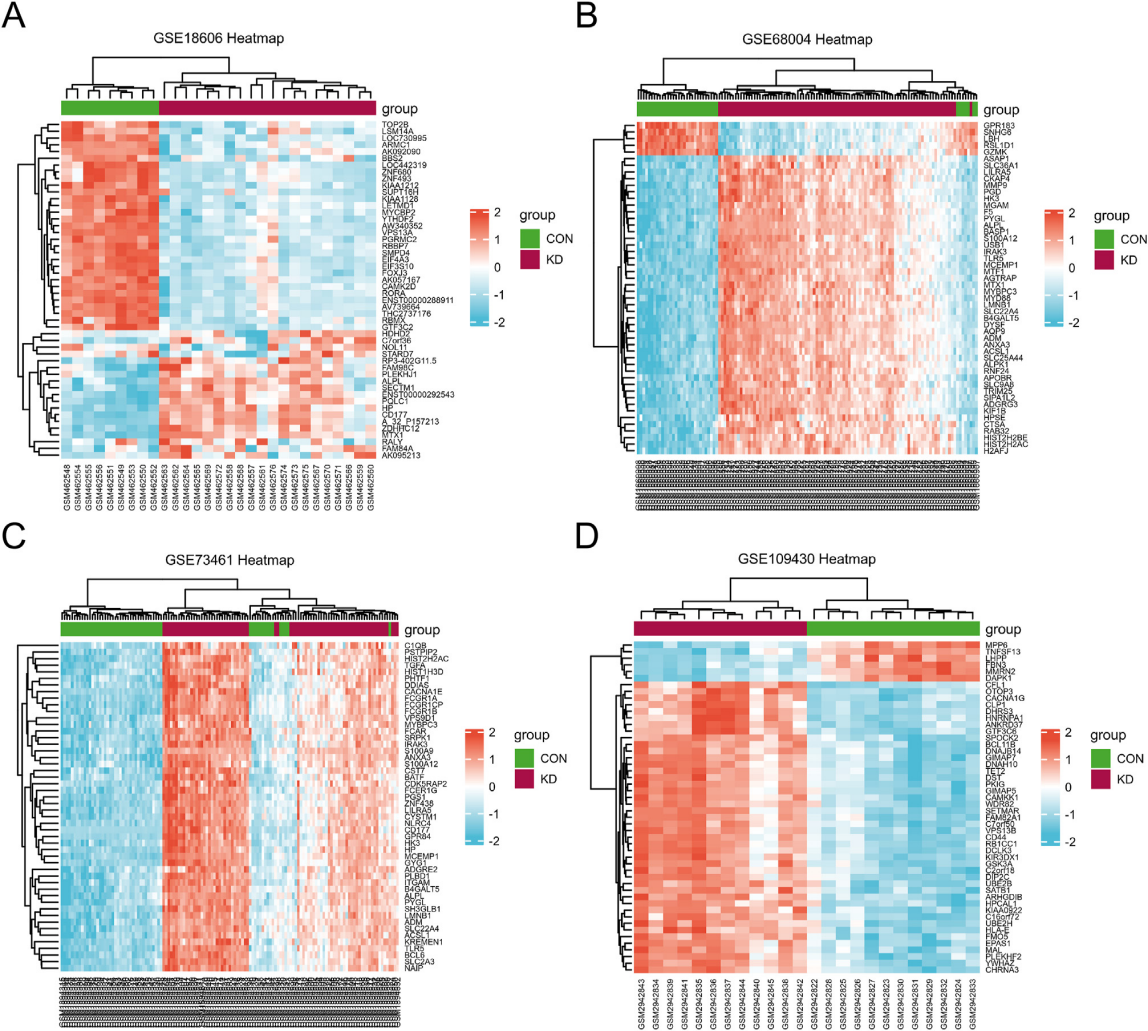
Clustered heat map of the top 50 DEGs and DMGs. (A, B and C) The heat map of top 50 DEGs in GSE18606, GSE68004, and GSE73461; (D) The heat map of top 50 DMGs in GSE109430.

The study design flowchart was shown in Figure 1. R software was used to analyzed each microarray data to acquire the DEGs or DMGs, separately. In three gene expression profiling datasets, 23 genes were up-regulated (2068 in GSE18606; 2667 in GSE68004, 1436 in GSE73461) while 74 genes were down-regulated (1885 in GSE18606; 2352 in GSE68004, 1467 in GSE73461). In gene methylation profiling dataset, there were 451 hypermethylated genes and 2408 hypomethylated genes. Finally, we identified 43 hypermethylated, low-expressing genes (Figure 2A) and 12 hypomethylated, highly expressed genes (Figure 2B). The clustered heat map of GSE18606 (Figure 3A), GSE68004 (Figure 3B), and GSE73461 (Figure 3C) (with the top 50 DEGs, |logFC| > 1.0) and the top 50 DMGs (|δβ| > 0.2) of GSE109430 (Figure 3D) were shown.

### 3.2 Functional and pathway enrichment analysis

We conducted GO enrichment analysis for the two groups of MDEGs, respectively (Table 1). For hypermethylated and low expressed genes, BP and CC analysis results were mainly associated with immunity. KEGG pathway enrichment analysis results in T cell-related items, including T cell receptor signaling pathway, Th17 cell differentiation, PD-L1 expression and PD-1 checkpoint pathway in cancer, Th1 and Th2 cell differentiation, and Primary immunodeficiency. For the hypomethylated and high expressed genes, the BP analysis was also associated with immunity.

**Table 1:** GO and KEGG analysis of MDEGs.

Ontology	ID	Description	Count	Adjust P value	Q value
**Hypermethylation and low expression**					
BP	GO:0042110	T cell activation	40	8.6339E-19	7.682E-19
BP	GO:0030217	T cell differentiation	27	4.7221E-15	4.2015E-15
BP	GO:0050851	antigen receptor-mediated signaling pathway	30	4.7221E-15	4.2015E-15
BP	GO:0050852	T cell receptor signaling pathway	25	4.7221E-15	4.2015E-15
BP	GO:0030098	lymphocyte differentiation	30	6.5772E-14	5.8521E-14
BP	GO:1903039	positive regulation of leukocyte cell-cell adhesion	24	2.2634E-13	2.0139E-13
BP	GO:0022409	positive regulation of cell-cell adhesion	25	7.1695E-13	6.3791E-13
BP	GO:0050870	positive regulation of T cell activation	22	3.7867E-12	3.3693E-12
BP	GO:0007159	leukocyte cell-cell adhesion	27	5.8876E-12	5.2385E-12
BP	GO:0002429	immune response-activating cell surface receptor signaling pathway	31	1.3063E-11	1.1623E-11
BP	GO:1903037	regulation of leukocyte cell-cell adhesion	25	2.6521E-11	2.3597E-11
BP	GO:0022407	regulation of cell-cell adhesion	28	4.339E-11	3.8606E-11
BP	GO:0045785	positive regulation of cell adhesion	28	4.339E-11	3.8606E-11
BP	GO:0050863	regulation of T cell activation	25	4.339E-11	3.8606E-11
BP	GO:0046631	alpha-beta T cell activation	17	3.2382E-10	2.8812E-10
BP	GO:0051251	positive regulation of lymphocyte activation	24	1.0701E-09	9.5212E-10
BP	GO:0002696	positive regulation of leukocyte activation	25	2.4414E-09	2.1723E-09
BP	GO:0050867	positive regulation of cell activation	25	5.035E-09	4.4799E-09
BP	GO:0051249	regulation of lymphocyte activation	27	1.4483E-08	1.2886E-08
BP	GO:0033077	T cell differentiation in thymus	11	1.6368E-07	1.4563E-07
BP	GO:0046632	alpha-beta T cell differentiation	12	7.3366E-07	6.5277E-07
BP	GO:0045058	T cell selection	9	8.2554E-07	7.3453E-07
BP	GO:0031295	T cell costimulation	9	3.9825E-06	3.5434E-06
BP	GO:0031294	lymphocyte costimulation	9	4.4833E-06	3.989E-06
BP	GO:0070661	leukocyte proliferation	18	5.171E-06	4.6009E-06
BP	GO:0046651	lymphocyte proliferation	17	7.3149E-06	6.5085E-06
BP	GO:0032943	mononuclear cell proliferation	17	7.834E-06	6.9703E-06
BP	GO:0046635	positive regulation of alpha-beta T cell activation	9	8.2089E-06	7.3038E-06
BP	GO:0050671	positive regulation of lymphocyte proliferation	12	9.3446E-06	8.3143E-06
BP	GO:0032946	positive regulation of mononuclear cell proliferation	12	9.8355E-06	8.7512E-06
BP	GO:0045061	thymic T cell selection	6	1.696E-05	1.509E-05
BP	GO:0070665	positive regulation of leukocyte proliferation	12	1.774E-05	1.5784E-05
BP	GO:0050856	regulation of T cell receptor signaling pathway	7	4.5729E-05	4.0687E-05
CC	GO:0009897	external side of plasma membrane	22	1.4915E-06	1.4671E-06
CC	GO:0001772	immunological synapse	8	1.9313E-06	1.8997E-06
KEGG	hsa04660	T cell receptor signaling pathway	14	6.7487E-08	6.0417E-08
KEGG	hsa04659	Th17 cell differentiation	14	6.7487E-08	6.0417E-08
KEGG	hsa05235	PD-L1 expression and PD-1 checkpoint pathway in cancer	12	5.7009E-07	5.1036E-07
KEGG	hsa04658	Th1 and Th2 cell differentiation	12	6.2887E-07	5.6298E-07
KEGG	hsa05340	Primary immunodeficiency	8	3.2887E-06	2.9441E-06
**Hypomethylation and high expression**					
BP	GO:0042110	T cell activation	6	3.3633E-06	1.2252E-06
BP	GO:0050863	regulation of T cell activation	5	1.8235E-05	6.643E-06
BP	GO:0007159	leukocyte cell-cell adhesion	5	1.8235E-05	6.643E-06
BP	GO:0002696	positive regulation of leukocyte activation	5	2.3818E-05	8.6768E-06
BP	GO:0050867	positive regulation of cell activation	5	2.3818E-05	8.6768E-06

### 3.3 PPI network construction and hub gene identification

**Figure 4:**
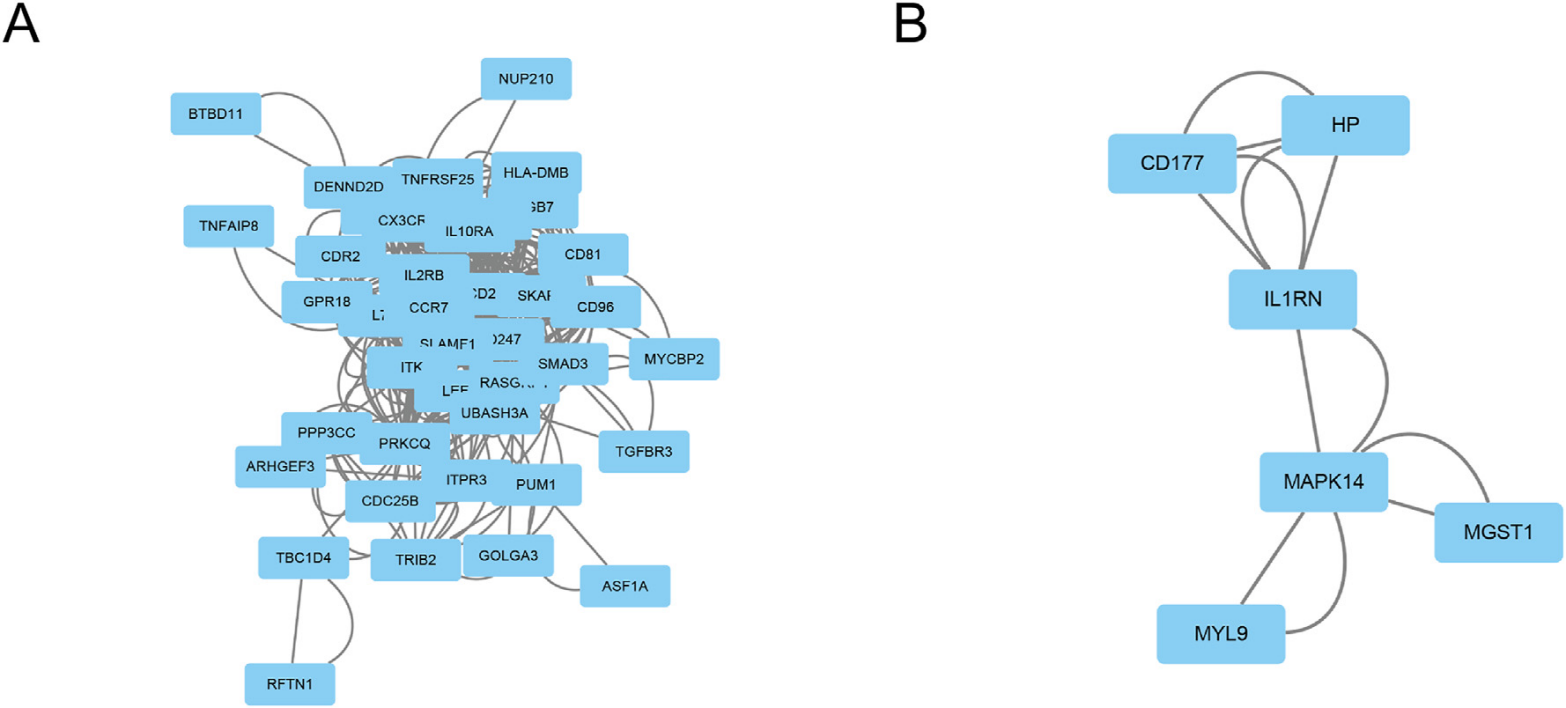
PPI network of MDEGs. (A) Hypermethylated and low expression genes; (B) Hypomethylated and high expression genes.

The dots in the PPI network represent different genes, and the lines between the dots represent the interaction between the genes. The number of lines linked to one gene was defined as the degree of connectivity of that gene. The gene with a high degree of connectivity was deemed as a hub gene with important biological functions. The PPI network was visualized using Cytoscape software, and the size of the nodes represents the connectivity degree. The hypermethylated and low expression genes PPI network was shown in Figure 4A, the hypomethylated and high expression genes PPI network was shown in Figure 4B. We using cytohubba to screen the hub genes. We used all the twelve ranking methods to rank the hub genes. We counted the genes that we get from these methods. Three hypermethylated and low expression genes (CD2, IL2RB, and IL7R) and three hypomethylated and high expression genes (CD177, IL1RN, and MYL9) were identified (Table 2).

**Table 2:** Hub genes among the MDEGs ranked in cytohubba

Hypermethylation and low expression genes	Count	Hypomethylation and high expression genes	Count
CD2	10/12	CD177	12/12
IL2RB	10/12	LI1RN	12/12
IL7R	10/12	MYL9	11/12

### 3.4 Module analysis

**Figure 5:**
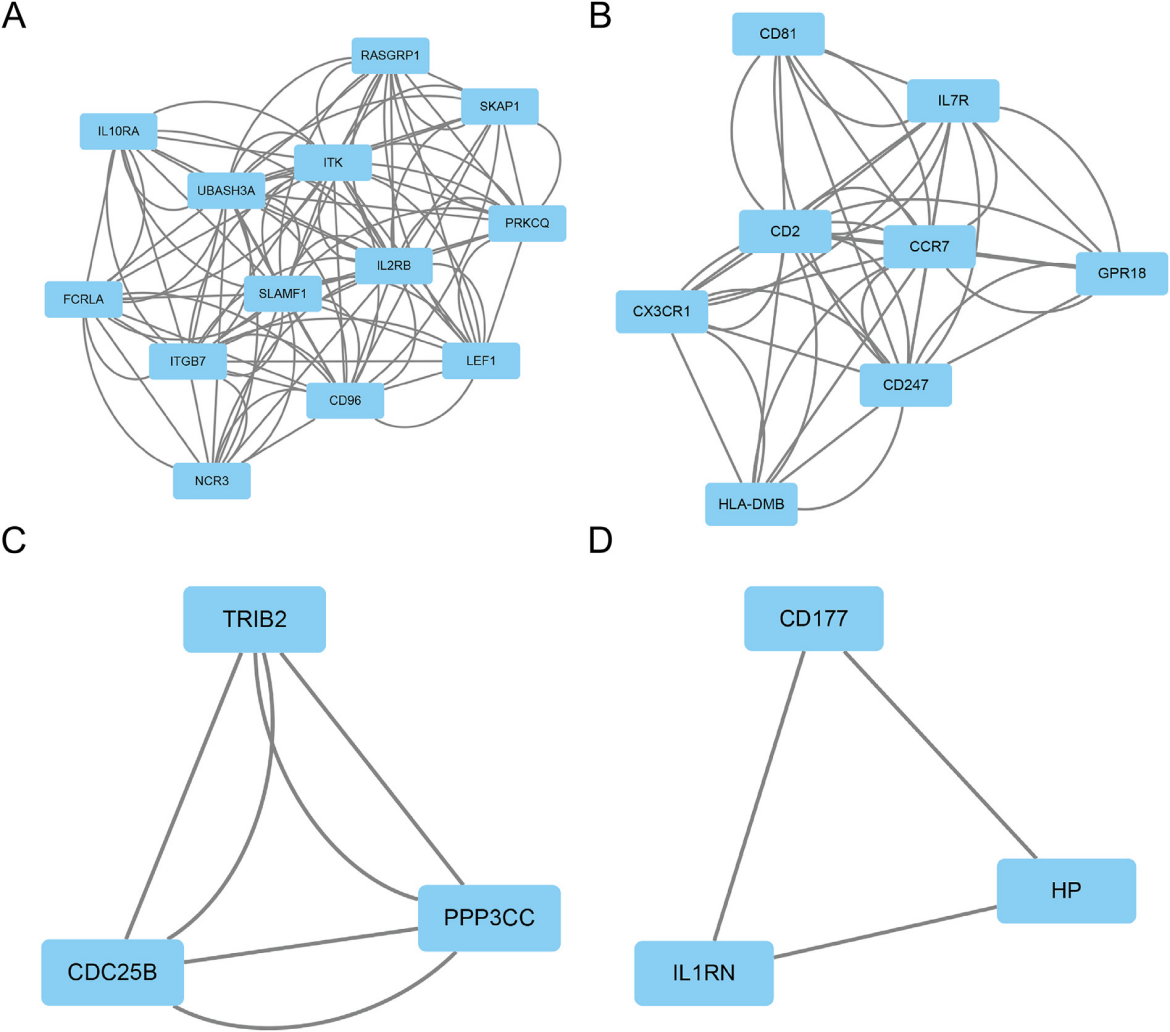
Module analysis of MDEGs. (A, B, and C) The hypermethylation and low expression genes in Module 1, 2, and 3; (D)The hypomethylation and high expression genes in Module 1.

Figure 5 shows that three modules in the hypermethylated, low expression genes network (Figure 5A-C) and one module in the hypomethylated, high expression genes network (Figure 5D) were statistically significant. In Table 3, we conducted the GO and KEGG enrichment analysis. The results indicated that the hypermethylated, low expression genes were enriched in immunity and hematologic related process and pathways.

**Table 3:** Module analysis of the PPI network.

Category	Module	Score	Nodes	Enrichment and pathway discerption	Genes
Hypermethylation and low expression	1	9.000	13	GO:0032633-interleukin-4 production GO:0002715-regulation of natural killer cell mediated immunity GO:0050852-T cell receptor signaling pathway GO:0002228-natural killer cell mediated immunity hsa04660-T cell receptor signaling pathway	ITK, PRKCQ, LEF1 RASGRP1, CD96, NCR3 ITK, PRKCQ, SKAP1, UBASH3A RASGRP1, CD96, NCR3 ITK, PRKCQ, RASGRP1
	2	6.286	8	GO:0042110-T cell activation GO:0050863-regulation of T cell activation GO:0007159-leukocyte cell-cell adhesion hsa04640-Hematopoietic cell lineage hsa04060-Cytokine-cytokine receptor interaction	CD2, CD81, CCR7, GPR18, HLA-DMB, IL7R CD2, CD81, CCR7, HLA-DMB, IL7R CD81, CCR7, CX3CR1, HLA-DMB, IL7R CD2, HLA-DMB, IL7R CCR7, CX3CR1, IL7R

### 3.5 Identification and validation of six hub genes

**Figure 6:**
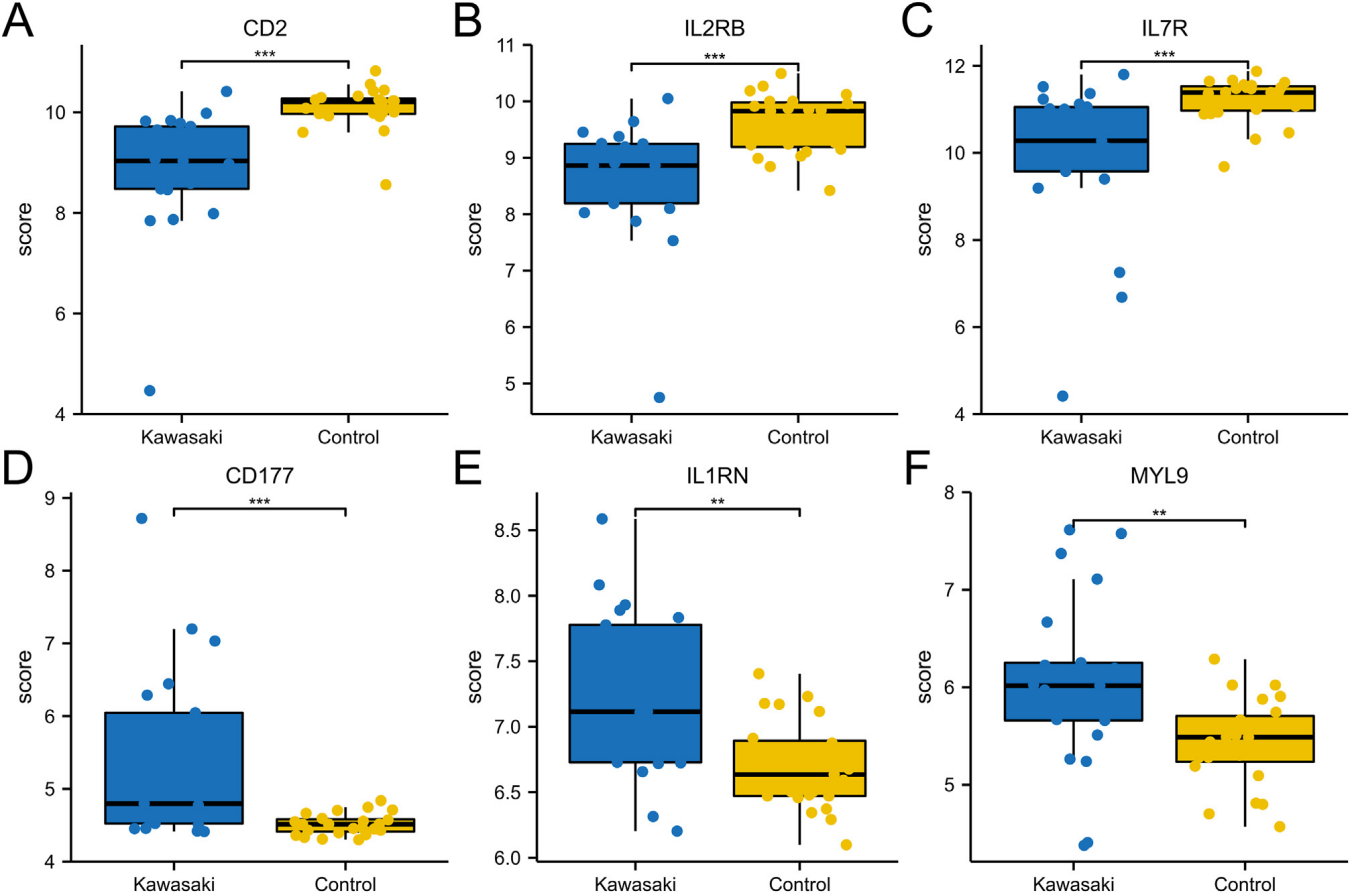
The expression of the six genes expression, using GSE100154 from the GEO database.

**Figure 7:**
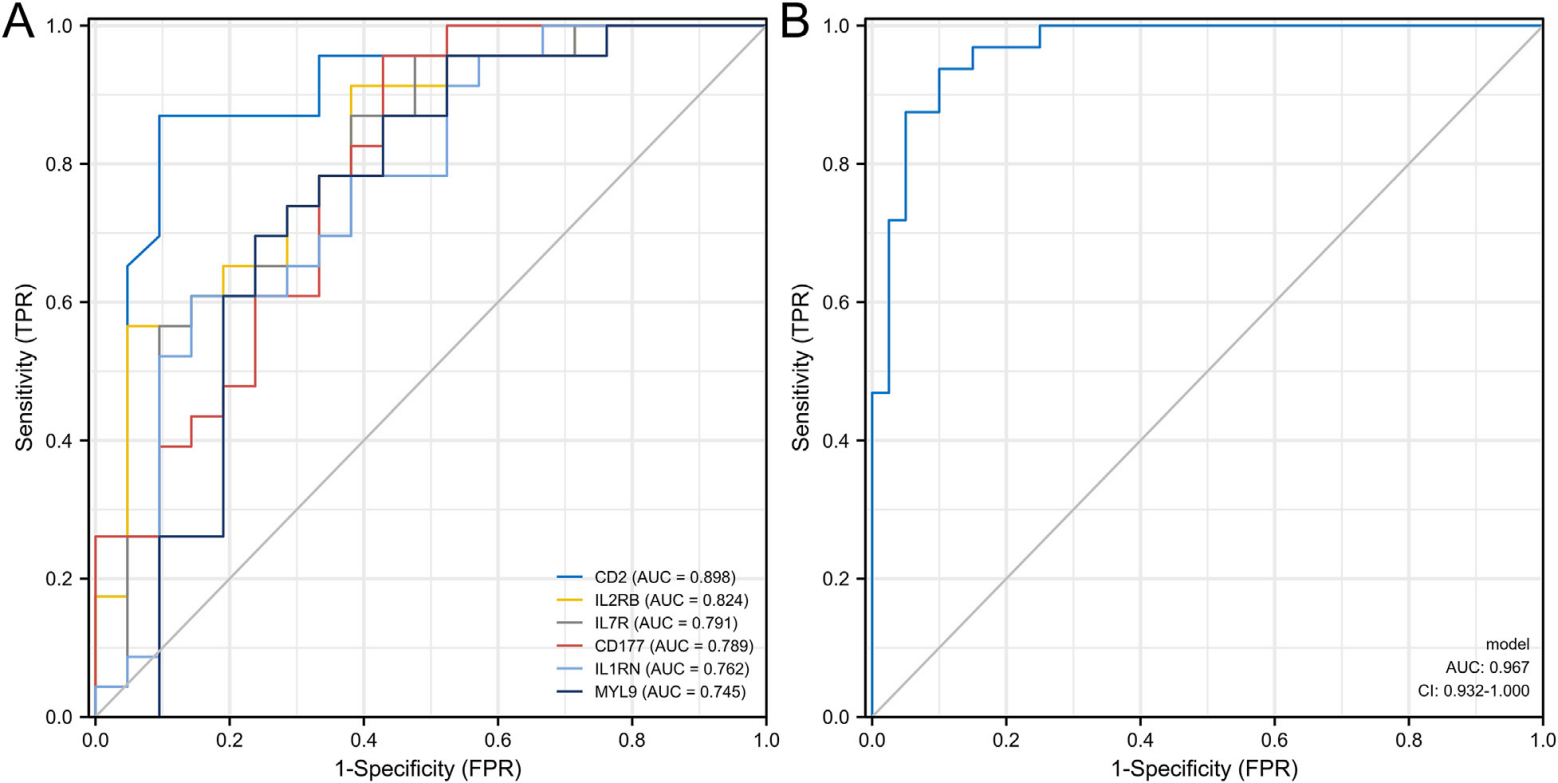
(A) ROC for six hub genes, respectively; (B) ROC for all the six hub genes.

Figure 6A-F showed the boxplots of six hub genes in GSE100154. We found that the expression of the three hypermethylated, low expression genes and the three hypomethylated, high expression genes were different between KD and healthy whole blood. Meanwhile, we have drawn the ROC for six hub genes, respectively, the AUC was from 0.745 to 0.898 (Figure 7A). We also draw the ROC for all the six hub genes, and the AUC was 0.967 (Figure 7B).

### 3.6 Establishment of diagnostic model

**Figure 8:**
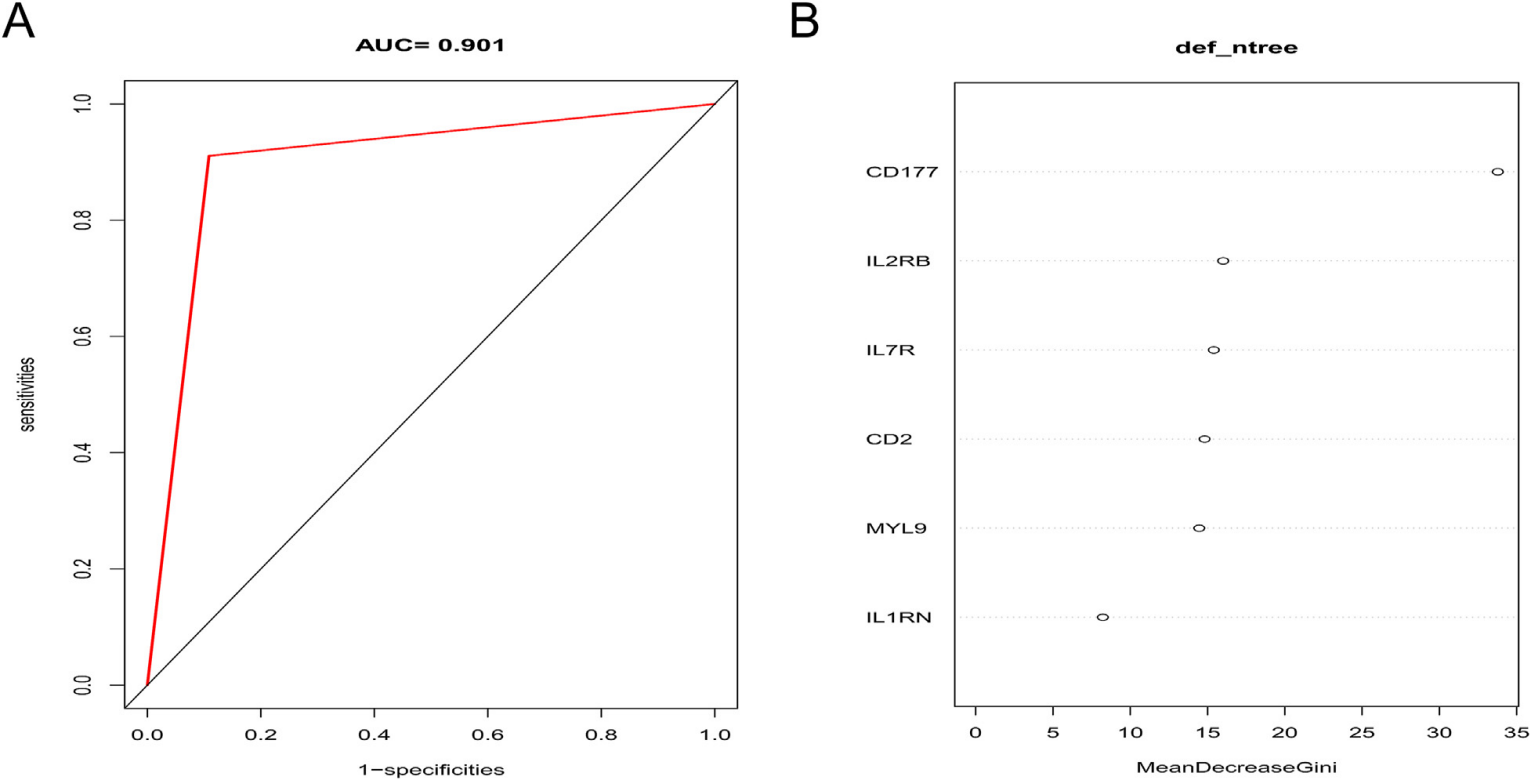
(A) ROC for test set of the diagnostic model; (B) Mean decrease Gini for diagnostic model.

We contrasted the diagnostic model by using six hub genes based on RF. All 332 samples were divided into training set (232 samples) and testing set (100 samples). The ROC of test set was shown in Figure 8A, AUC was 0.901. Mean decrease Gini was ranked (Figure 8B). CD177 (33.777222), IL2RB (16.012435), IL7R (15.407190), CD2 (14.807332), MYL9 (14.463159), IL1RN (8.227982). The accuracy was 91.30%, sensitivity was 0.891, specificity was 0.911.

## 4 Discussion

KD is the most common acquired heart disease, which onset under 5 years old. The cause of the disease is unknown [2]. Currently, the diagnosis of KD is mainly depended on non-specific clinical manifestations, but some patients with atypical clinical manifestations cannot be diagnosed and treated in time. This increases the risk of CALs, or even developing into a coronary aneurysm, by missing the optimal treatment period [3]. Therefore, we attempted to find potential genes related to the pathogenesis of KD through existing databases, and at the same time, to establish the clinical diagnosis model for KD. MDEGs between KD and healthy samples were screened by analyzed gene expression and methylation profile data of GEO database. In present study, we identified three hypermethylated, low expression genes and hypomethylated, high expression genes using bioinformatics analysis, separately. We verified the functional of these 6 potential hub genes. Meanwhile, we constructed a clinical diagnostic model to reduce the miss diagnosis rate of KD.

GO enrichment analysis showed that hypermethylated, low expression genes and hypomethylated, high expression genes were mainly enriched in immune-related BP, especially T cell-related BP. Hitata et al. [20] found that the number of γδT cells increased in KD patients. At the same time, studies have found that T cells in KD patients infiltrate on the coronary artery wall, and T cells can secret cytokines which can damage the elastic lamina and collagen fiber in coronary artery wall, and then causing or aggravate CALs [21, 22]. In contrast, Huijpers et al. [23] and Furukawa et al. [24] reported that T cells decreased in patients with KD, and the proliferation of T cells that TCR/CD3 dependent was inhibited. Meanwhile, in the acute phase of KD patients, T regulatory cells was decreased [25, 26]. These opposite studies can be confirmed in the GO enrichment analysis results obtained. Both the high and low expression genes were enriched in the T cell-associated BP, but number and degree of enrichment of the low expression genes were significantly higher than high expression genes. These results indicate that KD has a dual effect on T cells but mainly plays an inhibitory role. At the same time, since the expression of T lymphocytes in KD patients with CALs is up-regulated, we believe that the abnormality of hypomethylated, high expression MDEGs may be the reason for the occurrence of CALs in KD patients, but this still needs further study. Meanwhile, KEGG pathway enrichment analysis results in T cell receptor signaling pathway, Th17 cell differentiation, and Th1 and Th2 cell differentiation can also suggest that T cells is a key point of KD. Although hypermethylated, low expression genes are enriched in PD-L1 expression-related pathways, current studies on PD-L1 are limited to tumors, and there is no study related to KD [27].

PPI analysis showed that three hypermethylated, low expression hub genes were CD2, IL2RB, and IL7R. In memory T cells, CD2 expressed higher, and it can upgrade the activation of memory T cells [28]. In present study, CD2 was low expressed in KD patients, which may lead to decrease in T cells. IL2RB is a key point in T cell-mediated immune responses, and it can be saw in variety of immune diseases [29, 30, 31]. There is no study about correlation between IL2RB and KD, but there were evidences showed that IL2RB is important in T cells. Therefore, IL2RB may be an important gene in the pathogenesis of KD. IL7R is also a T cells related gene. Burns et al. [32] found that the level of IL7R in CD8+ T cells was increased in KD with CALs, but was absent in the majority of patients with KD in the acute stage. This is consistent with our findings that IL7R is down-regulated in KD patients. When IL7R levels rise, they are associated with an increased risk of CALs.

PPI analysis showed that three hypomethylated, high expression hub genes were CD177, IL1RN, and MYL9. CD177 is a neutrophil surface molecule, many studies have proved that there is a close relationship between CD177 and KD [33, 34, 35]. Huang et al. [33] found that, higher expression of CD177 was associated with intravenous immunoglobulin (IVIG)-resistant in KD. The mRNA profiling demonstrated that, in acute KD patients, the CD177 transcript higher in IVIG -resistant group compared to IVIG-sensitive group [34]. CD177 is an important gene in KD, other studies also indicated it is associated with IVIG-resistant [36, 37]. The high expression of IL1RN can significantly increase the susceptibility of KD. Wang et al. [38] conducted paired gene analysis on 221 KD patients and 221 control patients and found that the upgrade of IL1RN can increase the risk of KD. Wu et al. [39] also reached the same conclusion. The above studies are consistent with our previous studies. MYL9 has been studied mainly in cancer, but not in KD [40, 41].

Among all the six hub genes, IL7R, CD177, and IL1RN have been proved to be related to KD, while CD2 and IL2RN are related to T cells, which may be related to KD at the mechanism level. MYL9 was not found to be associated with KD. However, combined with our results, CD2, IL2RN, and MYL9 may play an important role in improving the onset risk of KD, which may be a direction of studying the pathogenesis of KD.

We verified these 6 hub genes in GSE100154 and found that the they had a good effect on the diagnosis of KD whether the AUC was calculated separately (0.745–0.898) or jointly (0.967). Therefore, we integrated four gene expression datasets and these six hub genes were used to build a random forest clinical diagnostic model. We compared the current model with other KD models. Huang et al. [42] constructed a KD diagnostic model with AUC = 0.906, sensitivity = 0.860, specificity = 0.805. Our AUC is similar to Huang’s, but sensitivity and specificity are higher than his. In Huang’s study, he found that his model was superior to the other five models. Therefore, we can argue that our current clinical diagnostic model of KD is superior to all existing models. In addition, we found that CD177 was the most important gene in the model, much higher than the other five genes. This suggests that CD177 maybe a key point of KD, which is consistent with the previous results.

## 5 Conclusion

Through systematic bioinformatics analysis, we studied the MDEGs in KD and healthy samples. We identified six hub genes that may play an important role in the progression of KD, and these 6 hub genes may also be important biomarkers for KD occurrence. The model established by us has a good effect on the diagnosis of KD, which can provide more accurate evidence for the diagnosis of KD and reduce the damage caused by missed diagnosis to patients.

### 5.1 Limitations of the study

The results of this study require further experiments.

## Declarations

### Author contribution statement

Hongxiao Sun, Changying Liu: Conceived and designed the experiments; Performed the experiments; Wrote the paper.

Xu zhang, Panpan Liu: Performed the experiments; Analyzed and interpreted the data.

Zhuanhui Du, Gang Luo: Analyzed and interpreted the data; Contributed reagents, materials, analysis tools or data.

Silin Pan: Conceived and designed the experiments; Wrote the paper.

### Funding statement

Dr. Silin Pan was supported by 10.13039/501100001809National Natural Science Foundation of China [81770316 & 81970249], Qingdao Science and Technology Plan [20-3-4-47-nsh], Taishan Scholars Program of Shandong Province [2018].

### Data availability statement

Data associated with this study has been deposited at GEO database.

### Declaration of interest’s statement

The authors declare no conflict of interest.

### Additional information

No additional information is available for this paper.
